# Incontinence of Urine in Children

**Published:** 1874-11

**Authors:** F. K. Bailey

**Affiliations:** Knoxville, Tennessee


### INCONTINENCE OF URINE IN CHILDREN.

BY F. K. BAILEY, M.D., KNOXVILLE, TENNESSEE.

While reading of late, in the *Obstetrical Journal of Great Britain and Ireland*, for August, 1874, some remarks by Dr. Henry Kennedy, upon incontinence of urine, I was led to reflect upon the occurrence of a few cases which have, at different times, come under my own notice.

It is not a common affection, but of a character which excites uneasiness in the minds of patients, and often baffles the skill of medical men. During the earlier months, the urine is voided at short intervals, and in a sense involuntarily. Like suckling, it it appears to be rather instinctive in common with voiding the rectum, to empty the bladder. The exact age at which a child begins to exercise a voluntary control over the sphincters is not well defined. This depends much upon habit formed by a proper attention on the part of the mother or nurse. We have all seen infants so trained, that at a surprisingly early age, they may be taught to evacuate the urine at stated times when held in a favorable position. That when a child thus taught, will seldom, if ever, soil its clothes or the floor. On the other hand we have all observed, while in the houses of those who are in other matters irregular and untidy, that their children grow up with filthy habits which are so disgusting to our sensibilities.

But, after all, the assiduous care which may be bestowed upon an infant in regard to its habits, and however young it may be taught to control the bladder, there are many that grow up with an inability to contain the urine. In other words, there is incontinence. This condition of things the medical man is often called upon to obviate or cure. Incontinence generally is found most frequent during sleep, though many are troubled at all hours, and while awake. Dr. K. has found it most common in boys, and that has been my own experience.

The pathological explanation of this affection is difficult to give. The sphincters are apt to be influenced by reflex causes. Mental conditions often cause a relaxation of the sphincters, both of the bladder and anus. We all have seen this to be true, not only in our species, but also in the lower animals. Were incontinence only found to obtain during sleep, we might maintain that the will was a great controlling agent in keeping the visical sphincter closed.

In seeking a remedy for this unpleasant affection, it is natural to make inquiry as to the general health. Are there worms in the rectum? If so, they should be removed. Sometimes the regulation of the amount of drink taken at night may be of service; but in too many cases the child will wet the bed after a most rigid scrutiny of condition, and the obviation of all apparent causes of a reflex nature. We generally conclude, under such circumstances, that the cause is nervous or spinal, and remedies in accordance with this view are employed.

In the early years of my practice, I gave cantharides with the best success. The tincture was prescribed in a small dose at first, and increased gradually, till strangury resulted or a cure obtained.

In cases where lytta was ineffective, I have used belladonna and nux vomica. Generally, however, when this first remedy did not relieve, the other failed. Perhaps one-half of all these cases where boys were the object of notice, were not benefited by any treatment, and a spontaneous cure was effected at puberty. How this occurred, I have never been able to explain satisfactorily, but some writer in the *Medical Journal*, whom I cannot locate and quote, has suggested that in many cases incontinence results from the reflex influence of phimosis, or an elongated prepuce, which is never retracted, or cannot be. Of the truth of this position, I am unable to judge from personal observation.

For this affection, when met with in girls, I have found lytta, or nux vomica, or both, to be curative, unless there are worms in the rectum, in which case their removal is insisted upon.

Generally, children of both sexes naturally outgrow this affection at puberty, as it is seldom that it continues after the age of fourteen or fifteen. It requires, oftentimes, close and persevering attention on the part of the practitioner to correctly diagnose cases of this kind. It is often very difficult to obtain all the facts, as children are very timid, and repel inquiry, besides being morbidly sensitive when the fact becomes known, and disinclined to communicate anything of their feelings. It is important that the physician should secure the entire confidence of the family, and impress them with the fact of his deep interest in the case, and a strong desire to remove the unwelcome ailment.

I have tried blistering the sacrum and perineal region occasionally, and cannot say that it will promise better than administering cantharides internally.

Sometimes, in little girls, there will be found an aphthous or herpetic affection within and around the genitalis, which may cause the trouble, and, of course, this should not escape notice.

The above thoughts are given as a result of observation during many years, in comparatively few cases.

Happily, a persistent, unyielding case of incontinence, is very rare, and in most cases a careful and intelligent investigation of all the facts will enable the physician to find a remedy.

Since writing the above, I have been able to call to mind a few cases:

1st. A boy of six or seven was brought to me in 1846 or '47. Small doses of tincture of lytta were given, and gradually increased. A cure was effected in a few weeks, and the trouble never recurred to my knowledge.

2d. A boy about the same age, in a neighboring town, the child of an acquaintance of the parents of No. 1. I prescribed for this case without seeing child, but considering that there was a similarity, gave the same remedy with a favorable result.

3d. A boy whose case was made known to me at about the age of ten. This was sometime in 1838, and I prescribed for him tr. lytta and other remedies, but with no effect. The trouble continued till the age of sixteen or more, but gradually stopped, and caused no further inconvenience in subsequent life. The cure was spontaneous.

4th. A girl of good general health began to be troubled at an early age, and came under my notice perhaps in 1845 or '46.

Nothing was of any avail that was given her, although diligently watched, but at puberty the trouble ceased spontaneously. She married young, and had two children prior to 1854.

5th. A boy of good general health, but small and exceedingly nervous in temperament, came under my notice about 1857 or '58, and was treated by means of tr. lytta, belladonna and nux vomica, and various other expedients were tried, with no benefit. Since puberty, there has been no trouble, and now he is a strong and healthy young man.

I have not met with a well marked case during the last ten or twelve years.

